# Exosomes secreted from human colorectal cancer cell lines contain mRNAs, microRNAs and natural antisense RNAs, that can transfer into the human hepatoma HepG2 and lung cancer A549 cell lines

**DOI:** 10.3892/or.2012.1967

**Published:** 2012-08-10

**Authors:** MITSURU CHIBA, MISAKO KIMURA, SAYA ASARI

**Affiliations:** 1 Department of Biomedical Sciences, Division of Medical Life Sciences, Hirosaki University School of Health Sciences, Hirosaki, Aomori 036-8564, Japan; 2 Department of Medical Technology, Hirosaki University School of Health Sciences, Hirosaki, Aomori 036-8564, Japan

**Keywords:** exosome, colorectal cancer, tetraspanin family, microRNA, natural antisense RNA

## Abstract

Exosomes are microvesicles that are released from various cells into the extracellular space. It has been reported that the components within exosomes vary according to the type of secreted cell. In the present study, we investigated the tetraspanin family proteins CD63, CD9 and CD81 as useful collection markers of exosomes derived from the three colorectal cancer (CRC) cell lines HCT-15, SW480 and WiDr. In addition, we aimed to detect the mRNAs, microRNAs and natural antisense RNAs within the exosomes secreted from the three CRC cell lines. Furthermore, we examined whether exosomes containing their RNAs were transferred into the hepatoma cell line HepG2 and lung cancer cell line A549. CD81 was detected in exosomes secreted from the three CRC cell lines. This result indicates that CD81 can be a collection marker of exosomes derived from the three CRC cell lines. When the RNA species within exosomes derived from the three CRC cell lines were examined, the mRNAs of housekeeping genes such as *ACTB* and *GAPDH*, the microRNAs such as miR-21, miR-192 and miR-221, and the natural antisense RNAs of *LRRC24, MDM2* and *CDKN1A* genes, were detected. We discovered their natural antisense RNAs within exosomes for the first time in the present study. Furthermore, PKH67-labeled exosomes derived from the CRC cell lines were taken up into HepG2 and A549 cells. These findings indicate that the intracellular RNAs enclosed within exosomes are secreted to the outside, and exosomes derived from the CRC cell lines are transferred into HepG2 and A549 cells. In conclusion, we reveal that exosomes derived from the CRC cell lines contain mRNAs, microRNAs and natural antisense RNAs, and can be delivered into HepG2 and A549 cells. These findings indicate that exosomal RNAs can shuttle between cells, and may be involved in the regulation of gene expression in recipient cells.

## Introduction

Exosomes are microvesicles (40–100 nm in diameter) of endocytic origin that are released from various cells into the extracellular space. In 1983, Pan and Johnstone were the first to show the presence of exosomes as secreted vesicles from sheep reticulocytes (1). It has been thought that exosomes are formed by the inward budding of multivesicular bodies (MVBs) and are released from the cell when intracellular MVBs fuse with the plasma membrane of the cell (2). Exosomes are present in cell culture supernatants as well as in body fluids such as serum/plasma, urine, amniotic fluid, and ascites fluid (2–6).

Exosomes are composed of a lipid bilayer, and they contain some proteins such as tetraspanins on the surface of their membrane (2). Tetraspanins comprise a large superfamily of cell surface-associated membrane proteins characterized by four transmembrane domains (7). There are 33 tetraspanin family genes in the human genome. These membrane domains control the proliferation and migration of cells through various cell adhesion and growth factor receptors (7,8). For example, it has been reported that TSPAN29 (CD9) is associated with ICAM-1, and TSPAN28 (CD81) is associated with CD19 (8–10). Among tetraspanin family proteins, CD63, CD9, and CD81 have been known to be located frequently on the surface of exosomes (2,11–13). Therefore, the tetraspanin proteins have been used as collection markers of exosomes. However, the appropriate collection markers of exosomes in each cell are not known because the components within exosomes vary according to the type of secreted cell.

The worldwide incidence of colorectal cancer (CRC) is high, particularly in developed nations. Recently, the development of useful biomarkers for the early diagnosis of CRC has been investigated. Microarray analyses have demonstrated that the differential expressions of mRNAs are observed between the CRC and normal colon tissues (14–18). In addition, it has been suggested that a large number of non-coding RNAs including microRNAs and natural antisense RNAs may be involved in the development of CRC (19,20). Valadi *et al* reported that the intracellular mRNAs and microRNAs are enclosed in exosomes and are secreted into the extracellular space (21). It has been suggested that RNAs within exosomes can be used as new diagnostic biomarkers because RNA components within exosomes differ according to the type and the physiological state of cells. Taylor *et al* identified microRNAs within exosomes to be as useful diagnostic biomarkers of ovarian cancer (22). Similarly, it has been suggested that the CRC cell lines secrete exosomes containing various RNAs and proteins, but the information on the type of components within exosomes is poor to date.

In the present study, we investigated the tetraspanin family proteins CD63, CD9, and CD81 as useful collection markers of exosomes derived from the three CRC cell lines HCT-15, SW480, and WiDr. In addition, we performed the detection of mRNAs, microRNAs, and natural antisense RNAs within exosomes secreted from these CRC cell lines. We also examined whether exosomes derived from these three CRC cells were transferred into the hepatoma cell line HepG2 and lung cancer cell line A549.

## Materials and methods

### Cell lines and cell culture

The human colorectal cancer cell line WiDr (JCRB0224), human hepatoma cell line HepG2 (JCRB1054), and human lung cancer cell line A549 (JCRB0076) were purchased from the Health Science Research Resources Bank (Osaka, Japan). The human colorectal cancer cell lines HCT-15 (CCL-225) and SW480 (CCL-228) were purchased from the American Type Culture Collection (ATCC, Manassas, VA). WiDr, HepG2, and A549 cells were cultured in Dulbecco’s minimum essential medium (D-MEM), which was supplemented with 10% fetal bovine serum (FBS), 100 U/ml penicillin, and 100 μg/ml streptomycin. HCT-15 and SW480 cells were cultured in RPMI-1640 medium, supplemented with 10% FBS, 100 U/ml penicillin, and 100 μg/ml streptomycin. Cell cultures were performed at 37°C in an atmosphere of 5% CO_2_.

### Isolation of exosomes from culture supernatants

The three CRC cell lines HCT-15, SW480, and WiDr were plated onto collagen-coated 10-cm dishes at a concentration of 1×10^6^ cells/dish using each of the media described above. After 48 h, the culture media were discarded, and the cells were washed three times in phosphate-buffered saline (PBS). Next, new media supplemented with 10% exosome-free FBS (by ultracentrifugation overnight) and the antibiotics mentioned above was added to the cells, and the cells were cultured. After 72 h, cell culture media were collected and sequential centrifugations were performed. Cell culture media were centrifuged at 300 × g for 3 min at 4°C to remove floating cells. These supernatants were then centrifuged at 2,000 × g for 15 min at 4°C, and at 12,000 × g for 35 min at 4°C to remove cell debris. These supernatants were then passed through a 0.22 μm filter. The filtrates were ultracentrifuged at 120,000 × g for 3 h at 4°C to collect exosomes using an Optima TLX Ultracentrifuge (Beckman Coulter, Brea, CA). Exosomal pellets were washed in PBS and were further ultracentrifuged at 120,000 × g for 3 h at 4°C. The final exosomal pellets were stored at −80°C until use or were resuspended in 100 μl of PBS.

### Measurement of exosomal protein concentration

Exosomal pellets were solved in 1% sodium dodecyl sulfate (SDS) solution and sonicated. The concentrations of exosomal proteins were measured using a Pierce BCA Protein assay kit (ThermoFisher Scientific, Wilmington, DE) and a Benchmark Microplate Reader (Bio-Rad, Hercules, CA) according to the manufacturer’s instructions.

### SDS-polyacrylamide gel electrophoresis (SDS-PAGE)

Exosomal proteins (10 μg) were mixed in equal volume with 2× sample buffer (0.125 mol/l Tris-HCl, 4% SDS, 20% glycerol, 10% 2-mercaptoethanol, 0.002% bromophenol blue, pH 6.8). These samples were boiled at 99°C for 5 min, and immediately cooled on ice. Electrophoresis of the exosomal proteins was performed using 10% Mini Protean TGX Precast Gels (Bio-Rad), Precision Plus Protein Dual Color Standards (Bio-Rad), and electrophoresis buffer (25 mM Tris, 192 mM glycine, 0.1% SDS, pH 8.3) at 200 V and 0.03 A for 30 min.

### Exosomal protein staining

Exosomal proteins in the loading gel were stained with Bio-Safe Coomassie brilliant blue (CBB) G250 Stain (Bio-Rad) according to the manufacturer’s instructions. Stained gels were photographed using a GS-800 Calibrated Densitometer (Bio-Rad) and a Quantity One software (Bio-Rad).

### Western blotting

The exosomal proteins of electrophoresed gels were transferred to Hybond-P membranes (GE Healthcare, Buckinghamshire, UK) using transfer buffer (25 mM Tris, 192 mM glycine, 0.01% SDS, 20% methanol, pH 8.3) at 100 V and 0.2 A for 60 min. Membranes were blocked in TBST buffer (25 mM Tris-HCl, 150 mM NaCl, 0.05% Tween-20, pH 7.2) containing 5% non-fat milk for 60 min at room temperature. After blocking, membranes were incubated with each primary antibody in TBST buffer containing 5% non-fat milk overnight at 4°C. As primary antibodies, 1:200 dilution each of anti-CD63 (sc-15363; Santa Cruz Biotechnology, Santa Cruz, CA), anti-CD9 (ab92726; Abcam, Cambridge, UK), and CD81 (ab79559; Abcam) were used. Membranes were washed five times for 5 min with TBST buffer and incubated for 60 min at room temperature with anti-rabbit horseradish peroxidase (HRP)-linked antibody (GE Healthcare) or anti-mouse HRP-linked antibody (GE Healthcare) at 1:5000 dilution prepared in TBST buffer containing 5% non-fat milk. Membranes were washed five times for 5 min with TBST buffer. Bound antibodies were visualized by chemiluminescence using an ECL Plus Western Blotting detection system (GE Healthcare). Luminescent images were analyzed using a ChemiDoc XRS (Bio-Rad) and the Quantity One software (Bio-Rad).

### Isolation and detection of exosomal RNAs

Exosomal RNAs were extracted using exosomal pellets and an ISOGEN II (Nippon Gene, Tokyo, Japan) according to the manufacturer’s instructions. Concentrations of exosomal RNAs extracted were examined using a Quant-iT RiboGreen RNA Reagent and kit (Life Technologies, Carlsbad, CA) and a Fluoroskan Ascent (ThermoFisher Scientific) according to the manufacturer’s instructions.

To confirm the size of the exosomal RNAs derived from the three CRC cell lines, exosomal RNAs were electrophoresed using an Agilent 2100 Bioanalyzer (Agilent Technologies, Foster City, CA) and an Agilent RNA 6000 Pico kit (Agilent Technologies) according to the manufacturer’s instructions. One nanogram of each of the exosomal RNAs were used for analyses.

### Reverse transcription-polymerase chain reaction (RT-PCR)

To obtain cDNAs derived from the mRNAs within exosomes, exosomal RNAs (1 ng) were used for the reverse transcription reaction. Reverse transcription reactions were performed using High Capacity cDNA Reverse Transcriptase kits (Life Technologies) according to the manufacturer’s instructions. These synthesized cDNAs were subjected to PCR in a 45 μl reaction mixture containing 1× buffer, 1.5 mM MgCl_2_, 0.1 mM of each dNTP, 0.025 U/μl of BioTaq HS DNA polymerase (Nippon Genetics, Tokyo, Japan), 0.5 μM of the primer pairs (Table I) and the cDNA template. PCRs were performed using a Veriti 96 Well Thermal Cycler (Life Technologies) under the following conditions: 7 min at 95°C, followed by 40 cycles each of 95°C for 30 sec, 60°C for 30 sec, and 72°C for 30 sec and a final elongation step at 72°C for 5 min.

In order to synthesize cDNAs derived from microRNAs within exosomes, reverse transcription reactions were performed using exosomal RNAs (1 ng), a TaqMan MicroRNA Reverse Transcription kit (Life Technologies), and TaqMan MicroRNA Assays (Life Technologies) according to the manufacturer’s instructions (Table II). PCRs were performed using a TaqMan Universal PCR Master mix II (Life Technologies) and a StepOne Plus Real-Time PCR system (Life Technologies) under the following conditions: 10 min at 95°C, followed by 40 cycles each of 95°C for 15 sec and 60°C for 60 sec.

To synthesize cDNAs derived from natural antisense RNAs, reverse transcription reactions were performed using exosomal RNAs (1 ng), the forward primers shown in Table III, and an AMV Reverse Transcriptase (Promega, Madison, WI) (19,23,24). PCRs were performed using cDNAs derived from natural antisense RNAs, a Power SYBR Green Master mix (Life Technologies), the primer pairs described in Table III, and the StepOne Plus Real-Time PCR system (Life Technologies) under the following conditions: 10 min at 95°C, followed by 40 cycles each of 95°C for 15 sec and 60°C for 60 sec.

The PCR products obtained above were electrophoresed using 4% agarose gels. Detection of amplified fragments was achieved by ethidium bromide staining using the ChemiDoc XRS (Bio-Rad) and the Quantity One software (Bio-Rad).

### PKH67 labeling of exosomes and their uptake into HepG2 and A549 cells

Exosomes derived from the three CRC cell lines were isolated as described above. They were then washed by being resuspended in PBS and ultracentrifuged at 120,000 × g for 3 h at 4°C. Exosomes were labeled using PKH67 Fluorescent Cell Linker kits (Sigma-Aldrich, St. Louis, MO) according to the manufacturer’s instructions, with minor modifications in the washing process. The washed exosomal pellets from the 100 ml culture media were resuspended in 700 μl of Diluent C (exosomal solution). PKH67 dye (1 μl) was diluted in 250 μl of Diluent C (PKH67 solution). Then, 250 μl exosomal solution and 250 μl PKH67 solution were mixed in a 4.7 ml centrifugation tube. Samples were mixed gently for 4 min, and 4.2 ml of 1% BSA was added to bind the excess PKH67 dye. PKH67-labeled exosomes were ultracentrifuged at 120,000 × g for 3 h at 4°C using the Optima TLX Ultracentrifuge (Beckman Coulter). Exosomal pellets were washed three times in PBS by ultracentrifugation. Finally, PKH67-labeled exosomes were resuspended in D-MEM or RPMI-1640 medium. As the negative controls, no PKH67 control and no exosome control were prepared. Exosomes were collected by ultracentrifugation without PKH67 dye, and then D-MEM or RPMI-1640 medium were added to the centrifuged tubes (no PKH67 control). After PKH67 dye was washed by ultracentrifugation without exosomes, the supernatant was discarded and media described above were added to the centrifuged tubes (no exosome control).

To examine the uptake of exosomes into other cells, HepG2 or A549 cells were plated in 8-well chamber slides (1×10^4^ cells/well) using each medium. After 24 h, the slides were washed three times in PBS, and each medium containing PKH67-labeled exosomes or negative control samples was added into each well. Cells were cultured for 48 h at 37°C in an atmosphere of 5% CO_2_. After incubation, the slides were washed three times in PBS, and 4% paraformaldehyde solution then added to the slides. These were fixed for 10 min at room temperature. The slides were washed three times in PBS again. Nucleus staining was performed using a ProLong Gold Antifade Reagent with DAPI (Life Technologies) and the slide was covered with cover glass. Finally, the cells were visualized under a confocal laser scanning microscope LSM710 (Carl Zeiss, Oberkochen, Germany) under the same conditions.

## Results

### Detection of tetraspanins in exosomes derived from the three CRC cell lines

**Figure 1:**
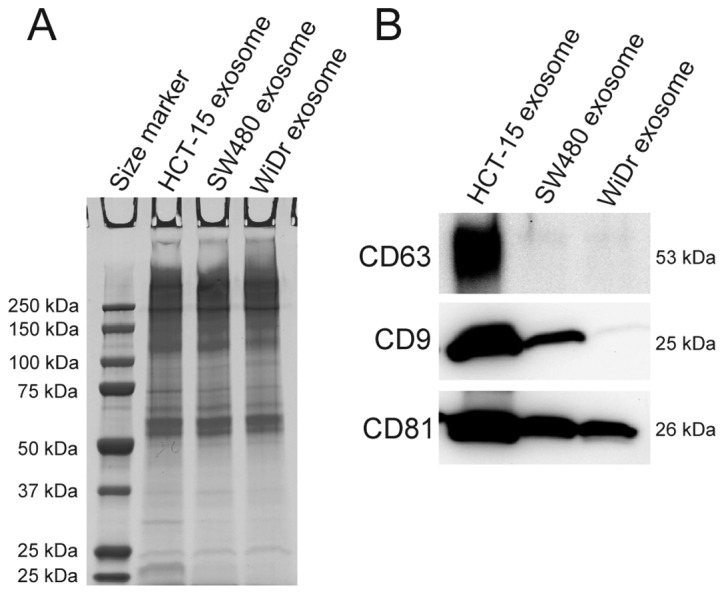
Detection of exosomal proteins derived from the three CRC cell lines. (A) Coomassie brilliant blue (CBB) staining of exosomal proteins derived from HCT-15, SW480, and WiDr cells. Protein contents in exosomes were similar between the three CRC cell lines. (B) Detection of CD63, CD9, and CD81 proteins in exosomes derived from HCT-15, SW480, and WiDr cells by western blotting. CD81 was detected in the exosomes derived from the three CRC cell lines examined.

It is known that exosomes contain various proteins, especially those of the tetraspanin family. CD63, CD9, and CD81 proteins were examined in exosomes derived from the three CRC cell lines HCT-15, SW480, and WiDr by western blotting. As shown in Fig. 1A, CBB staining showed that exosomes derived from the three CRC cell lines contained various proteins, and these proteins were similar in sizes with the exosomes of the three CRC cell lines. This finding suggests that the exosomes between the three CRC cell lines may have similar proteins. As shown in Fig. 1B, CD63 was detected in exosomes derived from HCT-15 cells; CD9 was detected in exosomes derived from HCT-15 and SW480 cells; and CD81 was detected in exosomes derived from HCT-15, SW480, and WiDr cells (Fig. 1B). These results indicate that exosomes derived from all the CRC cell lines examined contain CD81. Therefore, CD81 can be a collection marker of exosomes derived from the three CRC cell lines.

### Detection of exosomal RNAs in the three CRC cell lines

**Figure 2:**
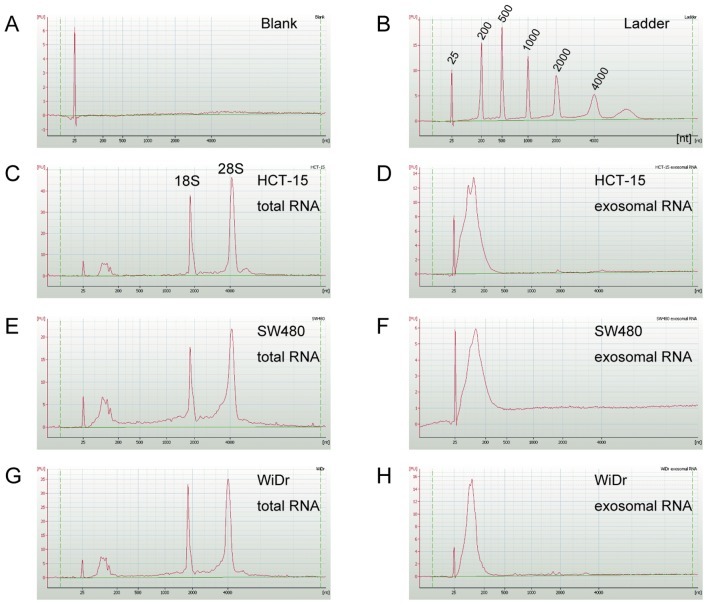
Detection of exosomal RNAs derived from the three CRC cell lines using an Agilent 2100 bioanalyzer. Data show the size distribution of exosomal RNAs and intracellular total RNAs derived from the three CRC cell lines. The peak detected ~25 nucleotides (nt) represents an internal standard. FU stands for fluorescence units. Exosomes derived from HCT-15, SW480, and WiDr cells contained small RNAs, and a number of exosomal RNAs were detected in the size range of 25–200 nt. On the other hand, 18S rRNA and 28S rRNA, which were detected in intracellular total RNAs, were rarely detected in exosomal RNAs. (A) Blank. (B) Ladder. (C) Total RNAs derived from HCT-15 cells. (D) Exosomal RNAs derived from HCT-15 cells. (E) Total RNAs derived from SW480 cells. (F) Exosomal RNAs derived from SW480 cells. (G) Total RNAs derived from WiDr cells. (H) Exosomal RNAs derived from WiDr cells.

To confirm whether exosomes derived from the three CRC cell lines contained RNAs, the exosomal RNAs were extracted using the ISOGEN II as described in Materials and methods. The size of exosomal RNAs was examined using the Agilent 2100 Bioanalyzer as described in the Materials and methods. As shown in Fig. 2, exosomes derived from HCT-15, SW480, and WiDr cells contained a large number of small RNAs, and their small RNAs were rarely detected in the size range: 25–200 nucleotides (nt).

**Figure 3:**
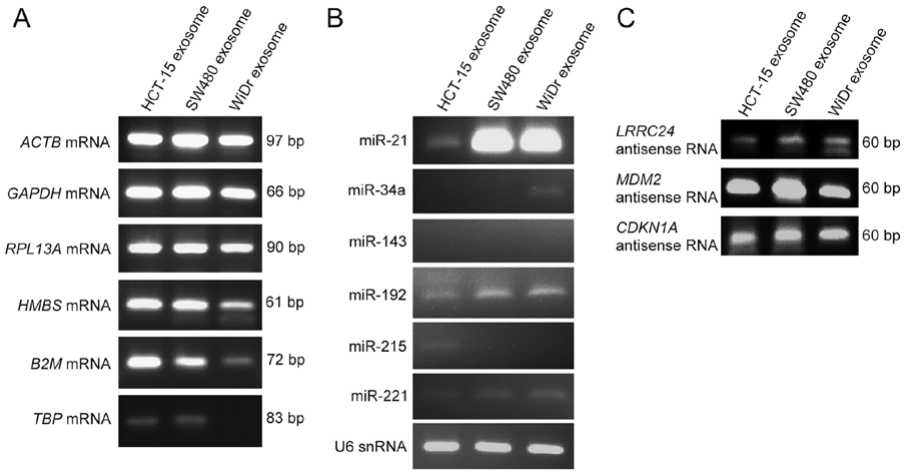
Identification of mRNAs, microRNAs, and natural antisense RNAs within exosomes derived from the three CRC cell lines by RT-PCR. The mRNAs, microRNAs, and natural antisense RNAs within exosomes derived from the three CRC cell lines were detected by RT-PCR as described in Materials and methods. (A) Detection of *ACTB, GAPDH, RPL13A, HMBS, B2M,* and *TBP* mRNAs. (B) Detection of miR-21, miR-34a, miR-143, miR-192, miR-215 and miR-221 as microRNAs and of U6 snRNA as small RNA. (C) Detection of the natural antisense RNAs of *LRRC24, MDM2* and *CDKN1A* genes.

In order to detect the mRNAs in exosomal RNAs, *ACTB, GAPDH, RPL13A, HMBS, B2M,* and *TBP* mRNA of housekeeping genes were examined by RT-PCR as described in Materials and methods. As shown in Fig. 3A, all the mRNAs examined above were detected from the exosomes derived from HCT-15 and SW480 cells; and the mRNAs, except for *TBP* mRNA, were detected from the exosomes derived from WiDr cells.

de Krijger *et al* stated that microRNAs such as miR-21, miR-34a, miR-143, miR-192, miR-215, and miR-221 were involved in CRC metastasis (25). Therefore, in order to detect their microRNAs within exosomes, RT-PCR of their microRNAs and U6 snRNA were performed as described in Materials and methods. As shown in Fig. 3B, miR-21, miR-192, miR-221, and U6 snRNA were detected from the exosomes of the three CRC cell lines, especially miR-21 was strongly detected from the exosomes of SW480 and WiDr cells; miR-34a was weakly detected from the exosomes of WiDr; miR-215 was weakly detected from the exosomes of HCT-15; and miR-143 was not detected from the exosomes of the three CRC cell lines.

Kohno *et al* reported that natural antisense RNAs of *LRRC24* gene was upregulated in the human CRC tissues, compared with the normal colon tissues (19). In addition, we detected the natural antisense RNAs of *MDM2* and *CDKN1A* genes in human cell lines in a previous study (23). Therefore, in order to examine whether natural antisense RNAs are present within exosomes, the natural antisense RNAs of *LRRC24, MDM2* and *CDKN1A* genes were examined by strand-specific RT-PCR as described in Materials and methods. As shown in Fig. 3C, all the natural antisense RNAs examined above were detected in the exosomes of the three CRC cell lines. We discovered that their natural antisense RNAs were within exosomes for the first time in the present study. These results indicate that the mRNAs, microRNAs, and natural antisense RNAs exist within the exosomes derived from the three CRC cell lines.

### HepG2 and A549 cells take up exosomes derived from the three CRC cell lines

**Figure 4:**
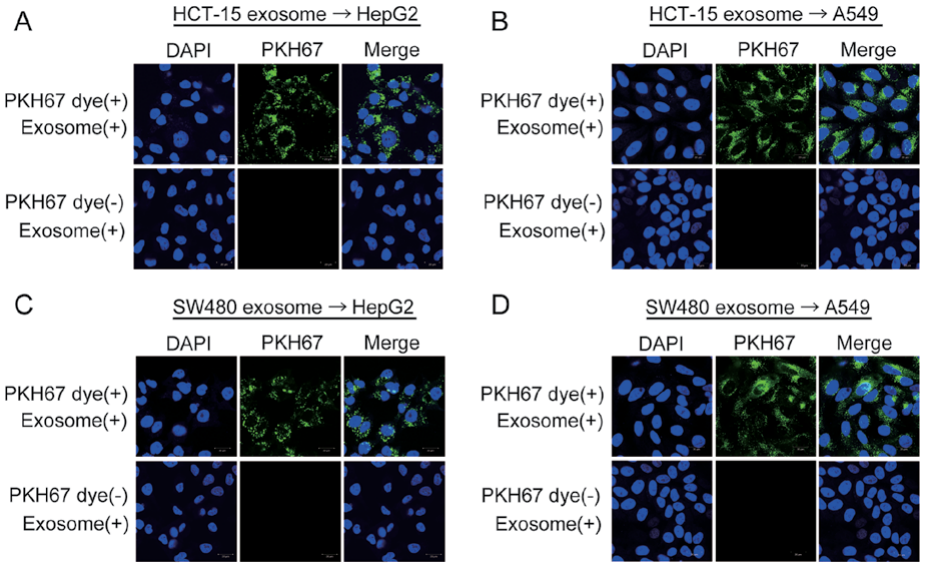

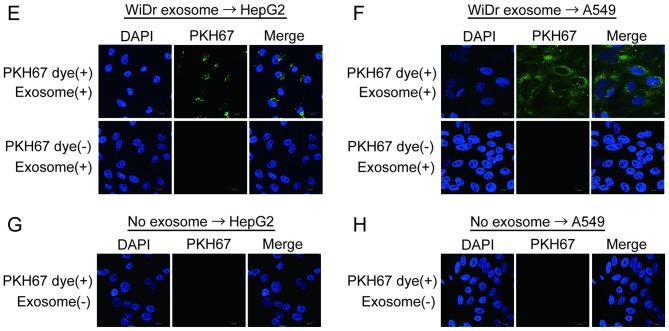
Uptake of exosomes derived from the three CRC cell lines into HepG2 and A549 cells. Exosomes derived from the three CRC cell lines were labeled with PKH67 dye (green fluorescence) as described in Materials and methods. PKH67-labeled exosomes were added to the culture media of HepG2 or A549 cells. Cells were visualized using a confocal laser scanning microscope (LSM710). (A and B) PKH67-labeled or non-labeled exosomes derived from HCT-15 cells were added to the culture media of HepG2 or A549 cells. (C and D) PKH67-labeled or non-labeled exosomes derived from SW480 cells were added to the culture media of HepG2 or A549 cells. (E and F) PKH67-labeled or non-labeled exosomes derived from WiDr cells were added to the culture media of HepG2 or A549 cells. (G and H) After PKH67 dye was washed by ultracentrifugation without exosomes, the supernatant was discarded. Then, D-MEM or RPMI-1640 medium were added to the centrifuged tubes. Their media were added to HepG2 or A549 cells (no exosome control).

In order to examine whether exosomes derived from HCT-15, SW480, and WiDr cells could be taken up in HepG2 or A549 cells, their exosomes were labeled with PKH67 dye (green fluorescence) as described in Materials and methods. When PKH67-labeled exosomes were added to the culture media of HepG2 or A549 cells, green fluorescence was observed in HepG2 and A549 cells using the confocal laser scanning microscopy (Fig. 4). Green fluorescence was not observed in the two negative controls (Fig. 4). These results show that exosomes derived from the three CRC cell lines are taken up into HepG2 and A549 cells, and that exosomes containing various RNAs can shuttle between cells.

## Discussion

In the present study, we showed that CD81 was an appropriate collection marker of exosomes derived from HCT-15, SW480, and WiDr cell lines using western blotting. We also demonstrated that exosomes derived from the three CRC cell lines contained the mRNAs, microRNAs, and natural antisense RNAs. Herein, we discovered natural antisense RNAs of *LRRC24, MDM2,* and *CDKN1A* genes within exosomes for the first time. Finally, we showed that exosomes derived from the three CRC cell lines were transferred into HepG2 and A549 cells.

Recently, the identification of exosomal RNA and protein components have been performed by a number of researchers. Valadi *et al* discovered various mRNAs and microRNAs within exosomes derived from mouse and human mast cell lines by microarray analyses for the first time (21). Montecalvo *et al* detected microRNAs within exosomes derived from immature and mature dendritic cells by microarray analyses (26). Those studies showed that RNA components within exosomes differed according to cell type. Herein, we examined the types of exosomal marker proteins and RNAs within exosomes derived from the three CRC cell lines. By detection of the tetraspanin family within exosomes derived from the three CRC cell lines by western blotting, CD63, CD9, and CD81 were detected within exosomes derived from HCT-15 cells; CD9 and CD81 were detected within exosomes derived from SW480 cells; and CD81 was detected within exosomes derived from WiDr cells (Fig. 1B). These findings indicate that CD81 is the most useful exosome collection marker of these CRC cell lines. Interestingly, Petersen *et al* found that exosome production increased significantly after CD63-knockdown in B lymphoblastoid cells (27). A type of tetraspanin family and the quantity of such a family may be involved in exosome production.

We identified various RNAs within exosomes. As shown in Fig. 3, miR-21 was strongly detected within exosomes derived from the CRC cell lines, especially SW480 and WiDr cells. MiR-21 is the most common and highly upregulated microRNA in the CRC cell lines, and it inhibits the translation of PDCD4 protein (28). Overexpression of miR-21 induces the invasion, intravasation, and metastasis of the CRC cell lines by downregulation of PDCD4 protein (28). Taken together, these findings suggest that miR-21 within exosomes derived from the CRC cell lines may regulate the expression of target genes into HepG2 and A549 cells, and may induce various functions (e.g., invasion, intravasation, and metastasis) in those cells.

Natural antisense RNAs, which are transcribed from the DNA strand opposite to the sense strand, have been demonstrated to be involved in the control of gene expression in eukaryotes. Kohno *et al* discovered that the natural antisense RNA of *LRRC24* gene was upregulated in the human CRC tissues, compared with normal colon tissues (19). Furthermore, we detected the natural antisense RNAs of *MDM2* and *CDKN1A* genes in human B lymphoblastic cells in a previous study, and showed that their natural antisense RNAs were in the cytoplasm (23). In the present study, it was clarified that their intracellular natural antisense RNAs were enclosed within exosomes. Their natural antisense RNAs within exosomes derived from the CRC cell lines may also regulate the expressions of target genes such as *MDM2* and *CDKN1A* mRNAs into the cytoplasm of HepG2 and A549 cells.

It has been reported that exosomes contain various RNAs and are transferred into other cells. Valadi *et al* demonstrated that mouse proteins could be synthesized into human HMC-1 cells when exosomes derived from the mouse mast cell line MC/9 were added to the supernatants of the cultured human mast cell line HMC-1 (21). Zhang *et al* demonstrated that microvesicles derived from the human monocytic cells THP-1 delivered miR-150 into the human endothelial cells HMEC-1, and that the elevated level of exogenous miR-150 effectively reduced c-Myb expression and enhanced cell migration in HMEC-1 cells (29). Those findings suggest that the mRNAs and microRNAs within exosomes are delivered into different cells and can function in those cells. We revealed that exosomes containing the mRNAs, microRNAs, and natural antisense RNAs were transferred into HepG2 and A549 cells. The transferred mRNAs into those recipient cells may be translated to proteins, or the transferred microRNAs and natural antisense RNAs may regulate the expression of target mRNAs. Thereby, their exosomal RNAs may induce the development and malignancy of tumor cells.

It has been reported that ceramide plays an important role in the external secretion of exosomes (30,31). Ceramide is synthesized from sphingomyelin by the action of neutral sphingomyelinase 2 (nSMase2), and is a major component of exosomes. Kosaka *et al* reported that nSMase2 was involved in the external secretion of microRNAs within exosomes (32). It is thought that exosomes are secreted from a cell by the vesicular transport pathway involved in MVBs (2,11), but the detailed mechanisms are not completely understood. Further studies are required to: understand the mechanisms of exosome production via MVBs of the CRC cell lines; identify comprehensively the RNA species in the CRC cell lines; and examine whether the transferred RNAs in the CRC cell lines function in recipient cells such as HepG2 and A549 cells.

In conclusion, we revealed that exosomes derived from the three CRC cell lines contained the mRNAs, microRNAs, and natural antisense RNAs, and were delivered into HepG2 and A549 cells. These important and novel findings indicate that exosomes containing various RNAs can shuttle between cells, and may regulate the gene expression into recipient cells.

## Figures and Tables

**Table I:** Primer sequences for detection of mRNAs.

Primer name	Sequences	Size (mer)	PCR products (bp)
*ACTB*	F: 5′-CCAACCGCGAGAAGATGA-3′	18	97
	R: 5′-CCAGAGGCGTACAGGGATAG-3′	20	
*GAPDH*	F: 5′-AGCCACATCGCTCAGACAC-3′	19	66
	R: 5′-GCCCAATACGACCAAATCC-3′	19	
*RPL13A*	F: 5′-GCATGAGCTTGCTGTTGTACAC-3′	22	90
	R: 5′-CATGGGCGATGCCTGTAAC-3′	19	
*HMBS*	F: 5′-GAGAAGTCCAAGCAACAGC-3′	19	61
	R: 5′-CCTTCAGAACTGGTTTATTAGTAGG-3′	25	
*B2M*	F: 5′-CATGGTTGTGGTTAATCTG-3′	19	72
	R: 5′-GAGATAACACATCAAGTTTTATG-3′	23	
*TBP*	F: 5′-CAGTATTGCAGGACAGAATATATG-3′	24	83
	R: 5′-TTGTACAGAGTACTCTGAAGAAAG-3′	24	

**Table II:** The list of TaqMan microRNA assays.

Assay name	Assay ID
hsa-miR-21	000397
hsa-miR-34a	000426
hsa-miR-143	002249
hsa-miR-192	000491
hsa-miR-215	000518
hsa-miR-221	000524
U6 snRNA	001973

**Table III:** Primer sequences for detection of natural antisense RNAs.

Primer name	Sequences	Size (mer)	PCR products (bp)
*LRRC24*	F: 5′-TACGTTCGCACAGCTAGAGG-3′	20	60
	R: 5′-TTGATGACGAACATCTCGTGGC-3′	22	
*MDM2*	F: 5′-AGACAACCAATTCAAATGATTGTGC-3′	25	60
	R: 5′-CTCTTATAGACAGGTCAACTAGG-3′	23	
*CDKN1A*	F: 5′-TTGATTAGCAGCGGAACAAGG-3′	21	60
	R: 5′-TCCATAGCCTCTACTGCCA-3′	19	
